# Supplementary material for: Molecular Factors in PAD2 (PADI2) and PAD4 (PADI4) Are Associated with Interstitial Lung Disease Susceptibility in Rheumatoid Arthritis Patients
Source: Cells. 2023 Sep 8;12(18):2235. doi: 10.3390/cells12182235 (PMC10527441; doi:10.3390/cells12182235)
Supplement: Supplementary file 1 [file cells-12-02235-s001.zip › cells-2563030-supplementary.pdf]

Supplementary material

Table S1: AIMs polymorphisms used for the calculation of Eigenvectors.

Chr	*SNV*	*ZAP (n=60)*			*CEU (n=120)*			*D*	*Study group (n=867)*		
**No.**	**# rs**	**A1**	**A2**	**MAF**	**A1**	**A2**	**MAF**	**Δ**	**A1**	**A2**	**MAF**
1	rs4528122	T	C	0.067	C	T	0.142	0.792	T	C	0.338
1	rs986690	G	A	0.017	A	G	0.25	0.733	G	A	0.347
4	rs10516422	G	A	0.283	G	A	0.017	0.267	G	A	0.234
5	rs10515716	T	C	0.267	C	T	0.208	0.525	T	C	0.432
6	rs1878071	A	C	0.317	C	A	0.217	0.467	A	C	0.49
9	rs4084051	T	C	0.25	C	T	0.175	0.575	T	C	0.483
9	rs7853112	C	A	0.25	A	C	0.35	0.4	C	A	0.395
9	rs10511491	C	T	0.25	T	C	0.391	0.358	C	T	0.358
9	rs1039336	A	G	0.133	G	A	0.242	0.625	G	A	0.433
9	rs10116714	A	G	0.183	G	A	0.05	0.767	A	G	0.449
9	rs1980888	G	A	0.033	A	G	0.1	0.866	G	A	0.411
9	rs4743556	C	T	0.172	T	C	0.167	0.661	T	C	0.486
12	rs6487927	C	T	0.033	C	T	0.475	0.442	C	T	0.275
13	rs2147155	0	T	0	G	T	0.5	0.5	G	T	0.15

ZAP: Zapotecs, CEU: Utah Residents with Northern and Western European Ancestry, MAF: Minor Allele Frequency, A: allele1 and 2. D, delta value, obtained between MAF in CEU minus MAF in ZAP populations.

Table S2: Estimated pairwise comparison of F_{ST} index in study groups

	RA-ILD	*RA*
RA	0.32 (0.578)	-
CHS	0.34 (0.853)	0.24 (**0.043**)

RA: Rheumatoid Arthritis; RA-ILD: Interstitial lung disease associated to RA; CHS: clinically healthy subjects. Show index F_{ST} (p-value).

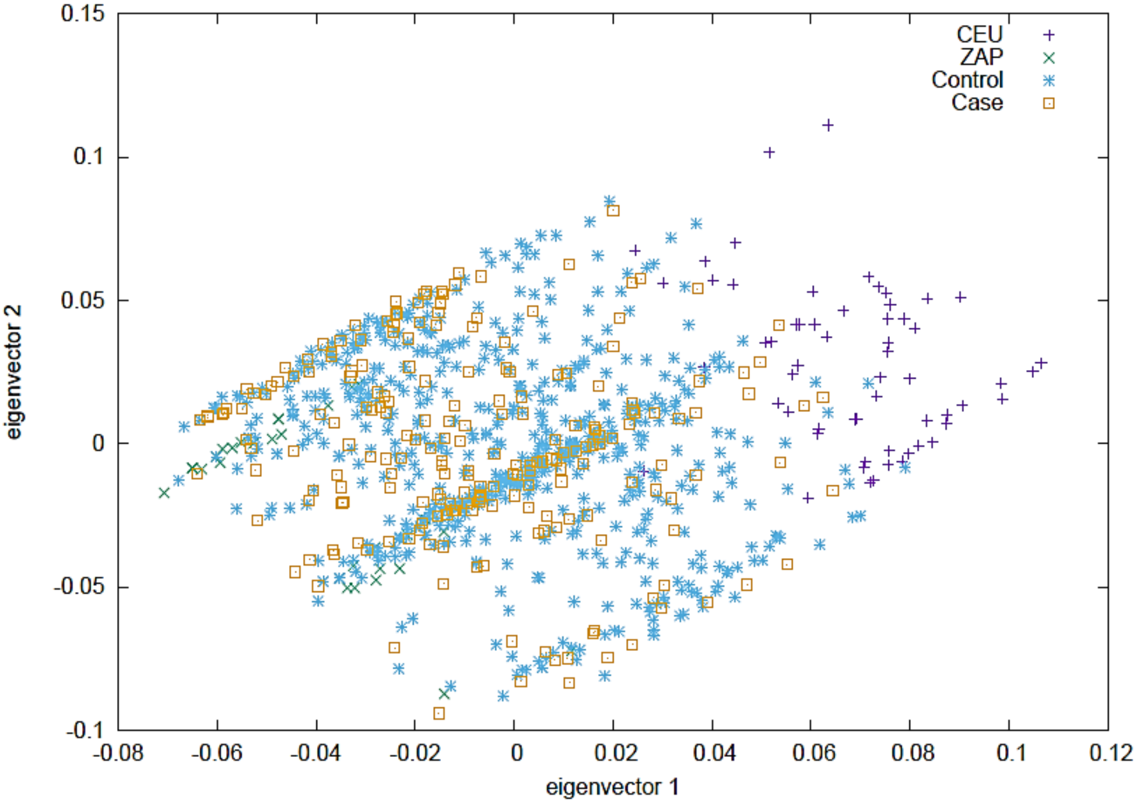

Figure S1: Principal component analysis, populations included in this study; the case group includes patients with RA-ILD and with only RA, the control group and the reference populations CEU and ZAP

Table S3: Demographic characteristics of subgroup of individuals whose PAD proteins were determined

	RA-ILD (n=34)	RA (n=31)	CHS (n=46)	p*
Age, years	59 (50-65)	55 (48.5-63)	53 (47-68)	0.049
Male, n (%)	5 (14.71)	2 (6.45)	24 (52.17)	NS
Smokers, n (%)	6 (17.65)	7 (22.58)	17(36.96)	-
Tobacco index	4.83 (2.9-6.9)	2.4 (1.7-5.8)	5 (3.3-7)	NS
Biomass-burning smoke, n (%)	10 (29.41)	6 (19.35)	7 (15.22)	NS
PAD2 (serum, ng/mL)	2.97 (1.85-5.52)	4.87 (1.5-9.39)	2.85 (1.51-4.66)	0.298
PAD4 (serum, ng/mL)	8.65 (0.88-35.34)	7.91 (1.29-43.2)	1.05 (0.27-2.67)	0.012
PAD2 (BAL ng/mL)	0.3 (0-15.57)	NA	NA	-
PAD4 (BAL ng/mL)	10 (5.76-35.28)			-

Quantitative variables are expressed in median (interquartile range) and categorical in number (percentage). RA-ILD: Interstitial lung disease-Rheumatoid Arthritis; BAL: Bronchoalveolar lavage. *Comparison between RA-ILD group and RA group.

Table S4: PAD levels with significant difference depending on genotype

Protein/gene	SNV-genotype associated	RA-ILD	RA	p
**PAD2/*PADI2***	rs2076615-AC	2.9 (1.8-5.6)	5.6 (2.1-9.9)	0.02
	rs1005753-TG	3.3 (1.5-5.5)	5.5 (2.9-14.9)	0.03
**PAD4/*PADI4***	rs11203366-GG	4.9 (0.4-39)	3.3 (1.4-24.4)	0.009
	rs11203367-TT	4.9 (0.4-39.1)	3.3 (1.4-29.4)	0.009
	rs11203367-CC	94.9 (19.8-134.3)	5.6 (2.4-22.4)	0.042
	rs1748033-TT	0.9 (0.33-126.7)	2.9 (0.3-15.1)	0.002
	rs874881-CC	6.8 (0.6-82.9)	2.8 (0.6-10.1)	<0.001

Median levels in ng/mL of PAD2 and PAD4 (Q1-Q3), p value in the comparison of RA-ILD *vs*. RA.

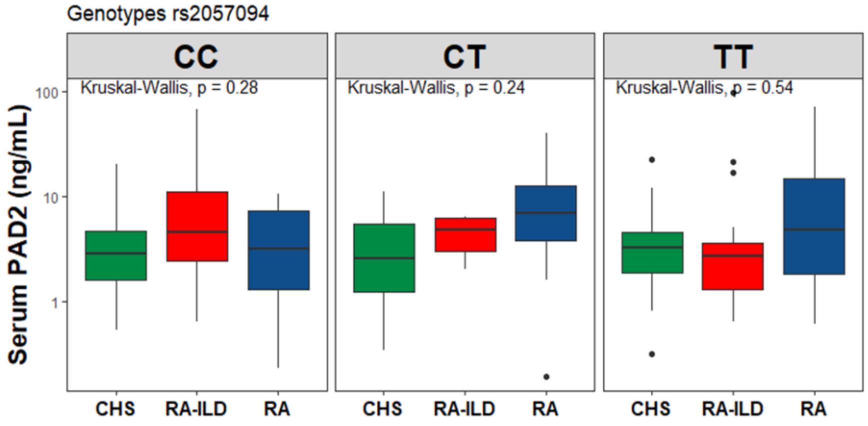

(a)

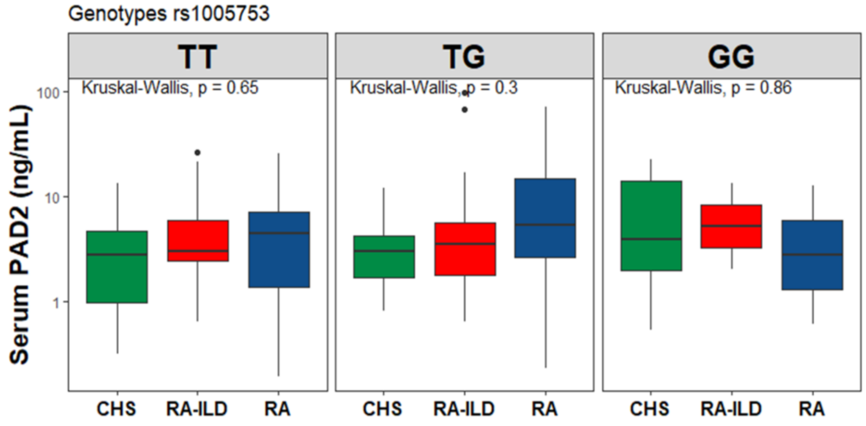

(b)

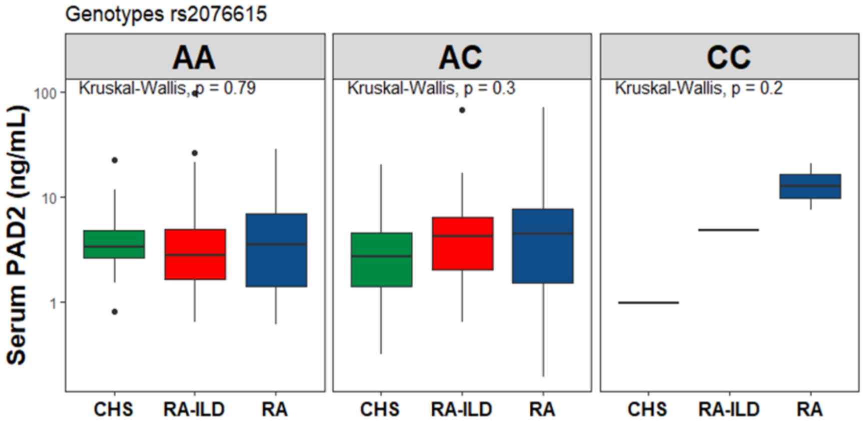

(c)

Figure S2: PAD2 levels compared to genotype of the SNVs in *PADI2* between study groups.
